# The Connected Community Pharmacy: Benefits for Healthcare and Implications for Health Policy

**DOI:** 10.3389/fphar.2018.01352

**Published:** 2018-11-28

**Authors:** Stephen Goundrey-Smith

**Affiliations:** SGS PharmaSolutions, Gloucestershire, United Kingdom

**Keywords:** interoperability, connected community pharmacy, electronic prescription service, summary care record, pharmacy services, healthcare

## Abstract

The need for interoperability of healthcare Information Technology (IT) systems in order to provide safe, efficient, and coordinated healthcare is universally recognized. Various health economies, such as the United Kingdom, the United States, and Australia, are seeking to develop regional, state-wide, or national systems of healthcare interoperability. In England, the community pharmacy network is a significant health provider, with important implications for provision of healthcare in deprived areas because of its accessibility. Historically, however, community pharmacies have operated on a silo basis, and have not shared information on their activities with, or been able to access information from, other National Health Service (NHS) healthcare providers. The development of services such as the Electronic Prescription Service and the Summary Care Record in England have helped to connect community pharmacy with the NHS infrastructure, and more comprehensive systems and datasets are proposed to integrate community pharmacy with the NHS in future. This paper will review the benefits of the connected community pharmacy, based on developments to date and reviewing evidence from other countries. It will describe some of the future developments that will support the connected community pharmacy in England, and discuss some of the implications for pharmacists and health policy makers.

## Introduction: the role of community pharmacy

Community pharmacy is an important primary care healthcare provider in England. There are 11,700 community pharmacies in England, and ~1.6 million people go to community pharmacy every day (Pharmaceutical Services Negotiating Committee, 2018). In England 89.2% of the population is likely to be only 20 min' walk from a pharmacy in most areas, and this can be as much as 99.8% in places of greatest need (Todd et al., 2014). Furthermore, pharmacists may usually be seen without an appointment. This level of access means that community pharmacies have potentially considerable impact on provision of health services in the most deprived areas of England. As well as supply of prescription and over-the-counter medicines, since the 2005 pharmacy services contract, community pharmacies have been increasingly providing clinically-focused enhanced pharmacy services such as Medicines Use Reviews (MURs), New Medicines Service (NMS) consultations, flu vaccinations, emergency contraception, health screening services, and many more.

Traditionally, in England, pharmacists in the community have been based at registered pharmacy premises, but there are now initiatives where pharmacists are working in new care settings, such as GP surgeries, care homes, and doing home visits. As experts in the actions, formulation and practical use of medicines, community pharmacists have an important role in advising on safe and effective use of medicines, providing services for medicines review, management of long-term conditions, and public health/screening services. Despite the increasing emphasis of community pharmacy on service provision, the dispensing workload for community pharmacy is increasing, possibly due to the increasing age of the population and the large number of specific medicines now available. Furthermore, service development takes time and resources, and there are challenges for pharmacists to ensure that they are adequately remunerated for the services they provide, and that their professional expertise in these areas is recognized, both by other healthcare professionals and by the public. The adoption of technology to support the expanding role of the community pharmacist is therefore critical, and this was highlighted by the Murray Report in 2016 (Murray, 2016).

Community pharmacies have been using technology to support their activities for over three decades now. The legal requirement for printed medicine labels, introduced in hospital pharmacies in 1976 (Anderson, 2005), led to the adoption of pharmacy computer systems, or patient medication record (PMR) systems, in community pharmacy during the 1980s. These systems were initially made available from pharmaceutical wholesalers, primarily as a tool to assist with ordering (and thus ensuring that orders were placed with the wholesaler); now, however, there are various IT suppliers providing licensed commercial solutions to meet community pharmacy needs, both for supply of medicines, and also provision of pharmacy services. These systems will maintain a record of medicines supplied to a patient, with appropriate decision support for supply, generation of labels, ordering and stock control, prescription endorsement and functionality to support a range of services such as MUR, NMS, flu vaccinations, supervised methadone, emergency contraception, and many more.

It is a common feature of healthcare IT in general that systems to support healthcare professionals often develop in professional and organizational silos (Goundrey-Smith, 2012). In hospitals, for example, a number of departmental systems have developed in silos—for example, the pathology results system, the pharmacy system, clinical scheduling etc—and, where this has happened, there has been a need to develop interfaces so that all the systems in the hospital can communicate. An alternative approach to system interoperability in hospitals has been to use a modular, whole-hospital single system such as Cerner Millennium, Epic, or Meditech. In this way, it has been possible to develop integrated electronic prescribing, medicines administration, and electronic discharge summaries for hospitals, which enables a connected hospital pharmacy.

Community pharmacy, however, is in a different position. Community pharmacies in England are distributed over a wide geographical area, are owned and managed by different organizations, have differing workloads and users. Furthermore, they utilize a range of pharmacy PMR systems, which have historically been adopted independently in each pharmacy, or group of pharmacies. Community pharmacy has not had the NHS support with IT infrastructure that general practice has had, and there is not the same conformance of requirements as with GP systems, which are managed under the GP Systems of Choice (GPSOC) framework (NHS Digital, 2018a). In Scotland and Wales, community pharmacy interoperability with the NHS has, in part, been addressed using a common infrastructure platform to which pharmacies can connect, but in England, with a larger number of community pharmacies distributed widely over a populous country, scalability, and organizational inertia have been significant barriers in the adoption of interoperability technology in pharmacies.

Historically, therefore, community pharmacies have been separate from other parts of the local NHS, and this has, in turn, been a barrier to integration of pharmacy professionals into the wider primary care team. There are a number of factors underlying community pharmacy's lack of connection with other local NHS providers—for example, organizational and professional differences, information governance and attitudes to data ownership—but lack of standards and infrastructure for electronic interoperability is a significant barrier and, if available, electronic interoperability would influence the other factors.

Yet, it is recognized in other contexts that there are potential benefits of integrated approaches to medicines optimization. A study by Scullin et al. (2012), in a US context, showed that the use an integrated system for medicines optimization has the potential for significant financial benefits and optimization of medicines for the healthcare provider.

Electronic interoperability would enable pharmacists to communicate electronically with GPs and other healthcare professionals and enable other healthcare providers in the NHS to communicate important information about medicines use to community pharmacists, and to refer patients with medicine-related issues to community pharmacists for medicines review, advice or, where appropriate, supply of an over-the-counter medicine. This connectivity would ensure that the community pharmacist's expertise in medicines is used fully and made more visible in the wider NHS. This has the potential to reduce the workload on GPs, and hopefully enhance the status and reputation of pharmacy professionals. The next section describes how electronic connectivity of the community pharmacy with other healthcare providers in England has developed so far.

## Community pharmacy connectivity: the story so far

The progress of connecting community pharmacy in England to the rest of the healthcare system has been slow but has gathered pace in recent years. In the early 2000s, pilots of electronic transfer of prescriptions (eTP) took place (Mundy and Chadwick, 2004), and this led to the development of the Electronic Prescription Service (EPS), within the NHS National Programme (NPfIT) under the auspices of NHS Connecting for Health at the time. The transfer of prescriptions electronically has been facilitated by infrastructure IT systems in various countries, such as the United States (Simonaitis et al., 2008), Sweden (Sjöborg et al., 2007), and Italy (Barbarito, 2006).

In England, EPS handles electronic prescription transfer from GP surgeries to community pharmacies. As well as handling the routing of electronic prescriptions into the pharmacy, EPS also enables the electronic submission of reimbursement claims to the NHS Business Service Authority. EPS is managed by NHS Digital (2018b), and handles in excess of 1 million transactions per day (Gooch, 2018).

The adoption of EPS has been slow. This has been, in part, due to the complexities of the total electronic prescribing environment and the challenges of evaluation of the system, as suggested by Lichtner et al. (2010) in their work on the EPS stakeholder map. In a survey of EPS use, Harvey et al. (2014) note that, in the original adoption of EPS, community pharmacists faced two challenges. The first was concerning missing prescriptions, which was disruptive to the pharmacy workflow, but which pharmacists considered a temporary issue, which could be remedied by minor modifications and user familiarity with the system. The second challenge was inherent design-related issues. which could only be surmounted by users employing unintended system work-arounds. This included, for example, printing out dispensing tokens to dispense from in order to release monitors for other pharmacy tasks, doing all dispensing with just one Smartcard, and problems with prescription endorsement and reimbursement claims. Some respondents to the survey stated that these situations arose because real-world users had not been consulted in the initial development of EPS. Harvey et al. conclude that these unintended uses and barriers would not have occurred had more user input taken place at an earlier stage in the design and implementation of EPS, but that system design modifications could still take place to resolve these issues. In another study published in 2014, Harvey et al. (2014) again note the challenge of missing prescriptions with the adoption of EPS but indicated that respondents still perceived EPS as helpful in streamlining pharmacy workflow. Many of the issues identified by Harvey et al have indeed been resolved as adoption of the system has progressed.

Historically, a key issue for community pharmacy professionals is that they have not had the information about a patient that is available to the GP—for example, medicines prescribed, not just dispensed, information on diagnosis, reason for prescribing, allergies, and adverse events—and they have not been able to communicate medicines-related issues electronically to the GP system. This is borne out by international research on information held in pharmacy systems. A key issue identified in research is the incompleteness of the dispensing record, when compared to the prescribing record. For example, reviewing the Danish prescribing and dispensing system, Glintborg et al. (2008) found that 6% of the medicines prescribed for patients were not listed in the dispensing record at the pharmacy, 27% of prescribed medicines were not mentioned by patients when visiting hospital and 18% of prescribed medicines were not mentioned by patients during home visits. In conclusion, the authors argued that both dispensing and prescribing histories should be used to prevent clinical error due to medicines not being viewable in the record. (Mabotuwana et al., 2009) compared the pharmacy dispensing record with the GP prescribing record and stated that a review of both records would be beneficial for the identification of medication adherence issues.

At the current time, pharmacies may not capture large amounts of patient data on the patient medication record system at the point of consultation, other than details of prescriptions dispensed and the patient's allergies. (Floor-Schreudering et al., 2009)looked at the content of the patient record in community pharmacies in Holland and noted that often the pharmacy record was not completed in any detail after the initial patient visit. As few as 67% of all prescription medicines and no OTC medicines were recorded. Furthermore, only 3.7% of allergies and drug intolerances were listed in the patient medication record in the pharmacy after the patient's initial visit. From this, they argued that pharmacists should try to collect as much of this information as possible at the outset.

Nevertheless, the patient's dispensing record may have information about medicines which cannot be obtained from other sources. Lau et al. examined the content of hospital medication records (Lau et al., 2000) and found that 25% of all prescription medicines taken by a patient were not recorded in the hospital medication record and that, for 61% of patients, one or more drugs being taken were not documented in the hospital patient record. Comparing these with community pharmacy records for the previous year, they concluded that pharmacy records may help to fill the gaps left by GP records and patient recollection.

The relatively low levels of patient data in pharmacy systems may have implications for what decision support functions can be provided by a pharmacy system. Rahimtoola et al. (1997) concluded that patient medication record systems in pharmacies provided little decision support on morbidity factors, such as contraindications, because coded disease-related data were not available to them. They therefore recommended that prescribers and pharmacists needed to share patient data.

For these reasons, pharmacists have, in the past, campaigned for “read-write” access to the GP record, in order for them to fulfill their role as healthcare providers. However, this has led to opposition from some GPs, and also concerns from some members of the public, fuelled by the popular press. Conversely, GPs have been interested in access to some aspects of the community pharmacy record that would complete their prescribing histories, for example, records of medicines dispensed and of pharmacy services, such as flu vaccinations administered in the pharmacy.

In the mid-2000s, the Summary Care Record (SCR) was developed, containing an outline patient history in a national record format, using information extracted from the GP system. The SCR contains details of medicines, allergies and adverse reactions, and significant medical history. With patient consent, additional information can be added to the SCR (NHS Digital, 2018c). The SCR was designed as a tool to support urgent, unscheduled care—for example, out-of-hours GP services and urgent hospital admissions. However, it has been found to be beneficial in the communication of the prescribing record and other important information relating to the use of medicines, from the GP system to pharmacy professionals. From around 2009, the SCR began to be used by hospital pharmacy staff for medicines reconciliation on hospital admission (Smith, 2009). Subsequently, a proof of concept study was done, looking at how the professional activities of pharmacists in the community in England and, as a result of that, a commitment was made by the government to roll the SCR out to community pharmacists in England in 2015 (National Pharmacy Association, 2018).

Another key element of the connected community pharmacy is the use of web-based pharmacy services applications. As mentioned previously, following the development of patient-centered pharmacy services since 2005, PMR suppliers developed functionality and modules to support these services, but the issue of how to refer information generated by these services to GPs still had to be resolved. Consequently, various web-based pharmacy services systems have been developed as tools to record pharmacy interventions, such as MURs and flu vaccinations, and then to transmit them to GPs. These include PharmOutcomes and Sonar Informatics solutions. These systems currently fulfill the important role of notifying GPs of services provided by pharmacies, such as flu vaccinations.

Systems have also been developed to facilitate transmission of information into pharmacies too. It is recognized that medicines discrepancies can occur when a patient is discharged from hospital (Michaelsen et al., 2015). Furthermore, the community pharmacist is in an ideal position to support a patient recently discharged from hospital on medicines related issues. However, although for many years, hospital systems have sent electronic discharge summaries to GPs, community pharmacists have not been in this communication loop. Consequently, the PharmOutcomes and the Refer-to-Pharmacy systems have been designed to route discharge summaries of patients discharged from hospitals to their local community pharmacy, so that the community pharmacist can: (a) resolve any issues concerning medicines changed during the hospital stay and (b) provide any appropriate pharmacy services for the patient—for example, an NMS consultation concerning a medicine commenced in hospital.

In addition to these, NHSMail provides a secure email service to enable communications containing patient information to be sent and received by healthcare providers in the NHS. While the system was originally designed for the individual named practitioner, it has now been developed to make it appropriate for use in community pharmacy—for example, introduction of group mailboxes–and it is now available for widescale use in community pharmacy (NHS Digital, 2017). However, at present, there is limited traffic in and out of community pharmacy via NHSMail, and it is not always monitored by GPs, so may not be the most efficient and reliable way for pharmacists to communicate with GPs. Nevertheless, use of NHSMail could be incentivized in future to increase its use. Another approach which is being adopted is the connectivity of the GP and pharmacist using a single system architecture. This approach has been developed by both EMIS with Pharmacy Access, and TPP System One with access to the system in community pharmacies in some locations.

To summarize, a number of systems are now available to enable the transmission of information in and out of the community pharmacy. These provide accurate prescription data to pharmacists and enhance the information that pharmacists have available to make professional decisions. These systems also facilitate the transfer of some information on professional services to the GP surgery. However, there is the potential for development of more comprehensive interoperability solutions for community pharmacy.

## Future developments

While community pharmacy in England is more connected than it was 20 years ago, there are many areas where more information flows could be developed. New types of information transfer could be facilitated and data transfers that already take place in some form could be done in a more consistent way.

In the past, health professionals have discussed “read-write” access to individual records—for example, with community pharmacy campaigns to access GP records. However, this approach may feel threatening for the health professionals who the data owner of the record—in this case, GPs. There is therefore a move away from this proprietorial approach toward the approach of connectivity through interoperability. Rather than specific records being accessed and written to, information is routed appropriately between the different systems, and record information is identified and made available appropriately to different users. This approach would facilitate two-way communications between GPs and pharmacy professionals, which is an important objective of “read-write access.”

Standards for clinical record content and format provide the basis for standard datasets for electronic interoperability and, in Great Britain, the Professional Records Standards Body (PRSB) is facilitating the development of the clinical standards that will underpin this interoperability (Professional Records Standards Body, 2018a). PRSB has developed standard headings for the hospital to GP electronic discharge record, and these headings have been mapped to Fast Healthcare Interoperability Resources (FHIR), so that they can be incorporated into electronic systems in a machine-readable way, according to an international convention. This will enable the hospital discharge dataset to be set up in a range of computer systems, mobile phones and devices, employing various database structures.

The PRSB standardization and FHIR curation process is now taking place for information flows from the pharmacy to the GP, and ultimately elsewhere in the NHS (Professional Records Standards Body, 2018b). Standard datasets are being created to capture and transmit information on various pharmacy services, including vaccinations, emergency supplies, MURs, NMS, and other pharmacy services.

Another important future development is the development of the NHS App, which will enable citizens to interact with NHS services on their mobile phones and devices (Department of Health Social Care, 2018). This will include functionality for ordering medicines, and this will have significant implications for community pharmacy, both in terms of interactions with patients, and professional relationships between pharmacies.

Furthermore, in the near future, pharmacies will need to implement the European Falsified Medicines Directive (FMD), which requires that medicines are verified at the point of supply to the patient, in order to identify and prevent use of falsified medicine packs (UK Falsified Medicines Directive Working Group, 2018). This applies to all prescription only medicines and will require medicines to have a 2D barcode scan before it is supplied. Hospital pharmacists are able to bulk scan all medicines to decommission them from the FMD system at the point they arrive in the hospital pharmacy. However, with community pharmacy, there is less flexibility, and the medicines must be decommissioned at the point of supply to the patient. This has created considerable complexity, given the unscheduled nature of collection of medicines from the pharmacy, the possibility of medicines not being collected or no longer required, and the challenge of scanning many medicine packs while the patient is waiting. It is likely that FMD will have a considerable implementation burden for community pharmacy and could have profound effects on pharmacy workload and workflow. FMD is often criticized that it is a huge business change with little tangible professional benefit for the pharmacist. The barcode data capture with FMD could be used for medicines safety deliverables, such as expiry date checking and batch recalls, but this is would be a future development.

In the past, the main critique of EPS and other systems that support community pharmacy was that they have been designed with only the pharmacist's supply role in mind, and do not take into account the active role of the pharmacist in receiving referrals, making recommendations to other healthcare professionals and advising the patient about medicines. It is to be hoped that, in future, infrastructure and datasets will be designed and implemented in a way that enables the full professional role of the pharmacist in patient-centered care to be demonstrated and contributed. The next section looks at the benefits that have been demonstrated with current systems that support a connected community pharmacy.

## Benefits of the connected community pharmacy

While there is little research on the benefits of using electronic systems in community pharmacy, in terms of safety and efficiency of workflow *within* the pharmacy, the development of systems that connect community pharmacies with other parts of the health service has led to research on benefits of these systems for both patients and professionals, and their impact on pharmacy practice and services. Various potential benefits have been identified with the available systems that connect pharmacy to the wider NHS.

The benefits of the eTP have been often articulated and discussed during the long adoption phase of EPS in England. These include:

- The electronic prescription transmitted is complete, legible and accurate, which reduces the possibility of errors and omissions in the medicine supply process. Nevertheless, the accuracy of the electronic prescription will be to some extent determined by the design of the GP system and the pharmacy patient medication record system. Previously, there have been instances where the dm+d code has been anomalous in EPS, or where the dosage instructions have been generated with Latin abbreviations, which have then been transferred to the label, and have confused the patient (Franklin et al., 2013). This problem has been, in part, dealt with by system usability and configuration changes, and the ultimate development of a standard for dose syntax will be a major development.
- The electronic prescription is transmitted securely (Mundy and Chadwick, 2003). It must be recognized, however, that the security of the electronic prescription is affected by the way it is displayed and stored in the pharmacy system. The pharmacy information governance procedures should address this issue. A previous UK survey of public, prescriber and pharmacist perceptions of eTP has described the security and confidentiality of patient information in the pharmacy setting, as a possible cause for concern (Porteous et al., 2003).
- Prevention of prescriptions that are unsigned, and therefore illegal (Franklin et al., 2013).
- Workflow efficiencies in community pharmacies. An eTP system may be helpful to community pharmacies in managing their repeat dispensing workload so that, for most patients, medicines are ready to collect from the pharmacy in a timely manner. A study of the England EPS has discussed the possible workflow benefits that the electronic transmission of prescriptions can provide (Harvey et al., 2011). These benefits can and should be used by community pharmacists to enable them to provide patient-focused services advice.
- Convenience to the patient in not having to submit a paper prescription to a pharmacy, and not having to wait for the dispensing process at the pharmacy. However, pharmacists will need to ensure their processes are streamlined to minimize waiting times. The effect of eTP on waiting times may well be affected in future by development such as: (a) availability of electronic prescriptions downloadable from a patient's mobile phone, or (b) the use of citizen driven prescription ordering, such as the NHS App.
- Electronic transfer of prescriptions opens up a means of communication between the prescriber and the pharmacist. The development of a comprehensive two-way means of communication between the prescriber and the pharmacist, would enable pharmacists to participate more fully in clinical care of the patient. Unfortunately, at the current time, the EPS in England only replicates the paper-based prescription system and does not enable significant two-way communication between pharmacists and prescribers, or between pharmacists and other clinical services.
- Electronic transfer of prescriptions has an effect on the medicines-related care processes in the health service, and therefore on professional roles and relationships in the system. In the United States, the eTP has affected the dynamics of the relationships between prescribers, pharmacies and health maintenance organizations (HMOs) (Fincham, 2009). As described earlier, the EPS system in England did not attempt to redesign the prescribing process, but to replicate the current paper-based system. However, analysis of the effects of widespread use in England of the current EPS suggest that the system has had effects on professional roles and management of risks, so will have had an impact on working relationships since its introduction.

The use of the Summary Care Record has been shown to have the following potential benefits (Jones, 2013):

- increased patient safety,
- improved clinical decision making,
- improved service efficiency and
- improved quality of care.

In the early stages of SCR roll-out, Greenhalgh et al. (2010) found that a key factor in accessing the SCR was the clinician's willingness or motivation to use it; in this study, clinicians cited use of available SCRs as between 0 and 84% of all opportunities. She found that access to the SCR enabled higher quality care and enhanced clinician confidence in some situations. However, use of the SCR did not necessarily reduce the length of a consultation or prevent onward referrals.

The use of the SCR in community pharmacy settings has been associated with the following benefits (NHS Digital, 2018d):

- allergies can be checked to prevent prescribing errors
- current medications prescribed can be checked for emergency supply purposes
- eligibility can be checked for services such as a free flu jab.

Although the SCR has been available in community pharmacy since 2015, its adoption in community pharmacy practice has been slow, and a quality payment system has been established to incentivize its use. Nevertheless, information from the SCR could be used in the following situations by community pharmacists:

- Making an emergency supply or out-of-hours supply. The SCR can be accessed to check the name, form, strength and dose of medication previously dispensed for the patient. This is especially important with patients who may be from outside the locality.

- During an MUR as a reference source for the patient's current medication, and to check their allergy status.
- For provision of an NMS consultation.
- Following a patient request, or minor ailments service (MAS) consultation, to confirm the appropriateness of any OTC medicines recommended, given the patient's prescribed medication.
- When dispensing a prescription medicine, to review the prescribing history or to inform a professional decision on an interaction, contraindication, or allergy. It is not yet known what added value the SCR might provide over the pharmacy's PMR system with routine dispensing.
- When an over the counter medicine is supplied, ideally potential drug interactions with prescribed medicines should be checked and the OTC supply record. However this is not usually possible in a busy community pharmacy.

A number of potential benefits have been discussed in relation to the use of web-based pharmacy services systems for routing hospital discharge referrals to community pharmacy in order for the pharmacist to provide a clinical service (transfer of care, or community pharmacy clinical handover):

1. Information about medicines for a patient discharged from hospital reaches their community pharmacy in a timely manner.
2. The pharmacist gets the discharge information at the same time as the GP.
3. Patients' questions about their discharge medicines are pre-empted.
4. Audit work (NHS Special Pharmacy Service, 2016) has shown that 6–13% of discharge summaries sent to GPs are not actioned appropriately. Being able to resolve these through community pharmacy medicines reconciliation is a key benefit, and is likely to have a beneficial impact on patient safety.
5. Issues arising from (3) and (4) are resolved in the community pharmacy, thus saving the GP time and reducing their workload.
6. Pharmacy contractual services are targeted to those patients who need them most, so saving the NHS money.
7. The skills of pharmacists and GPs are appropriately utilized.
8. Quality of care and patient experience are enhanced.
9. Messages can be sent to prevent unnecessary dispensing of previous regular medicines whilst the patient is in hospital (saving dispensing time and reducing medicines waste).
10. A clear purpose can be provided to the community pharmacist as to what to do with the information in each instance e.g., NMS, MUR, or stop providing a dispensing service (e.g., if a patient is moving to a care home serviced by another pharmacy).

Realization of some of these benefits has been demonstrated in the following research findings:

- Three-fold improvement on return on investment in discharge medicines due to reduced wastage and fewer episodes of subsequent unscheduled care (Hodson et al., 2014).
- 10% improvement in medicines adherence (Elliott et al., 2014).
- Significant reductions in rates of hospital readmissions and shorter hospital stays than patients who have not had a follow-up pharmacy consultation (Nazar et al., 2016).
- Reduction of community pharmacy dispensing time, and medicines waste (Gray, 2015).

Interoperable systems connecting community pharmacy with other healthcare providers in the NHS in England have the potential to increase the use of the community pharmacy as a hub of medicines optimization, and to ensure that the skills of pharmacists can be contributed fully to high quality patient care and demonstrated to the wider NHS.

It can be seen from the above findings that there are inherent benefits for patients if the community pharmacy is connected electronically into the wider healthcare system. These include higher quality care and a positive experience of using the service, in terms of convenience and a more patient-centered approach. Nevertheless, there is little research on patient attitudes to use of patient data in connected systems by pharmacists in the UK. A study of GP, pharmacist and patient attitudes to eTP, published in 2003 by Porteous et al. (2003) indicated that, although patients were positive about the potential convenience of the system, they had concern about pharmacists having access to parts of the patient record not relating to medicines. However, this work was done before the widescale adoption of EPS and the SCR in community pharmacy in England and there have been no published studies of patient experience relating to community pharmacy IT use since. This is unsurprising given that, when systems work well in the pharmacy, the patient should not be aware of their operation.

However, research is emerging to demonstrate that widescale deployment of electronic systems in community pharmacy has a cultural impact, as well as a business process impact. This should be of considerable significance to NHS managers and health policy-makers.

## Implications for policy makers

Information technology is often presented by both professional leaders and health policymakers as a way of improving clinical safety and mitigating risks. Petrakaki and colleagues note how, on the contrary, research has demonstrated that technology can lead to unintended consequences, and new clinical risks, risks which may develop through the interaction of technology and practice (Petrakaki et al., 2014). This phenomenon has already been observed in the adoption and use of hospital electronic prescribing systems, which have been described by Barber as “sociotechnical systems”—that is to say that the safe and effective operation of the entire system based on a combination of human and system design factors (Barber, 2010).

It should not be surprising that similar non-technical factors are at play within infrastructure systems used by community pharmacies. With the English EPS, Petrakaki et al. have demonstrated that, through a combination of technology and social context, risks may be socially constructed through the transformative capacity of the system and its ability to manipulate practice. These risks are then reinterpreted by the actors in the system across professional and cultural boundaries. Petrakaki et al. assert that this social construction of risk can facilitate the reordering of power and responsibility in social and professional relationships. This, they argue, has implications for policy makers, system designers and users (Petrakaki et al., 2011). I would also suggest that this systemic manipulation of power and responsibility has significant implications too for healthcare profession leaders, as it has the potential to transform both practice and professional roles.

Petrakaki and colleagues identified this issue specifically with community pharmacy in an earlier publication using data from a longitudinal study of EPS from 2009 to 2011 (Petrakaki et al., 2012). They found that, with EPS, the technology has the potential to shape the nature and values of pharmacy practice, professional roles, the degree of power professionals can exercise, their jurisdictions and professional boundaries. They assert that, where technology is introduced into a healthcare setting, it creates a system where there is an equilibrium of de-professionalization and re-professionalization for the professionals concerned.

This suggests that, in representing the interests of community pharmacy, pharmacy professional leaders should ensure that equilibrium of system design favors the professionalization—and therefore hopefully the professionalism—of community pharmacists, and that system implementers and health policy makers see the importance of pharmacy professionalism. The professional standing of community pharmacists is important for two reasons. Firstly, it will ensure that valuable pharmacy skills and knowledge are demonstrated and used in the provision of care, and this provides the value-added component for community pharmacy in a retail environment where remote purchase and automated, mass supply and fulfillment of goods is increasingly the norm. Secondly, it will ensure that the morale of pharmacy teams is maintained in a competitive environment, where patients and the public have increasingly high expectations of health professionals.

In their recent review of the development of electronic prescription systems in five selected countries—Denmark, Finland, Sweden, England, and the United States—Samadbeik et al. identify some of the elements of eTP implementation within a healthcare economy that should be the responsibility of the government and its policy-makers, via appropriate agencies (Samadbeik et al., 2017). They state that, in the light of past failures with such systems, the government should be responsible for facilitating safe and secure electronic transmission of prescription, as part of a national electronic healthcare infrastructure. They assert that the government should also provide legal and financial incentives for adoption of the system by stakeholders, to ensure that a critical mass of users is obtained. They also indicate that the eTP use case should be supported by message transmission standards and an interoperability framework. This suggests that, while there may be a case in England for greater incentivization of community pharmacists to adopt technology, provision of adequate and appropriate infrastructure requirements are rightly the duty of the government and its healthcare agencies. This is consistent with Sheikh, Atun, & Bates' call for independent evaluation of government-led health IT initiatives, in the light of evaluations of systems to date (Sheikh et al., 2014).

A similar socio-technical framework appears to be the case for the use of national care records, such as the Summary Care Record. Greenhalgh has noted that successful introduction of the SCR depends on the interaction between multiple stakeholders—clinical, political, technical, and commercial—who will have different perspectives, with different values, priorities, and ways of working (Greenhalgh et al., 2010).

An important counterpoint to the direction provided by governments and policy makers concerning the adoption and use of technologies is cultural attitudes to technology, which are formed by the attitudes of individuals toward the use of technologies, and their willingness to adopt them in their own life and work. The factors that affect this go beyond professional standards and the norms of professional practice, and encompass such things as psychological factors, perceived benefits of the use of technology in areas of life other than work (which is especially significant given the almost universal use of smart phones and devices in society at present), and patterns of adoption for any innovation, not just a technological one.

It should be noted that eTP, access to a shared care record, and systems that manage referrals to pharmacies and patient care outputs from pharmacies are just the basic systems for a connected community pharmacy. Other systems might include telehealth systems for remote consultations, mobile technology for adherence and therapy monitoring and, at the “high tech” end, smart infusion pumps. In theory, these also have profound implications for transformation of service delivery and professional practice. However, these systems are very much in their infancy, and experience is limited. Baines et al. (2018) have found that the numbers, design and quality of evaluation studies of these technologies are such that it is not possible at present to understand fully the benefits of these technologies, and for pharmacists to make appropriate decisions about their adoption. Baines et al point out that these technologies need considerable funding yet “despite the improvements in technology, there is limited evidence on how this translates to real settings and to consumer satisfaction.” It is likely that, due to the need for funding and adequate benefits assessment, the adoption of future technologies will need to be facilitated with a high level of intentionality by pharmacies, health service managers and policy-makers, if they are to be successful.

A recent study in Ireland looked at attitudes to the adoption of new service-enhancing technologies among community pharmacists (Sweeney C., 2018). The study found that community pharmacists were willing to actively engage with new technologies to deliver their services, although a number of barriers to technology implementation were identified—most notably, concerns about privacy & security, time available to implement technology due to work pressures, and pharmacists' lack of awareness of the full range of technologies that could be used. The authors noted that, in Ireland, there is a significant opportunity for community pharmacists to become early adopters amongst healthcare professionals, and to be positive agents for change.

## Conclusion: community pharmacy—the hub of medicines optimization

Reviewing the systems currently available, and their perceived benefits, together with planned future developments, the connected community pharmacy has considerable potential benefits for patients, healthcare professionals and policy makers. Some progress is already being made toward connecting community pharmacy electronically into the wider local and national healthcare system and thereby contributing to the integration of the community pharmacy service into the wider NHS. Nevertheless, future developments will enable a greater level of connection and communication between the community pharmacy in England with the wider primary care system.

## Recommendations: domains of the connected community pharmacy

In terms of professional goals and aspirations for community pharmacy, I propose five domains of activity that define the connected community pharmacy, and which should be supported by pharmacy interoperability technologies.

- Pharmacists provide a safe and effective supply chain for medicines.
- Pharmacists can receive referrals from other healthcare professionals.
- Pharmacists can assist patients and ensure appropriate medicines reconciliation following discharge from hospital and other transfers of care, for example between the person's home and a nursing/care home.
- Pharmacists can transmit details of their healthcare interventions and recommendations to other healthcare professionals.
- Pharmacists are able to interact with their patients to support pharmaceutical care and monitor therapy and adherence.

Work should continue to develop technologies that will support these five domains of professional activity for pharmacists. This will require input from, and communication between, health policy-makers and pharmacy professionals. An area of considerable development will be that of mobile technology and apps. At the time of writing, the use of these for medicines-related applications is at an early stage of development, yet these are likely to have considerable impact both on the working practices of health professionals and the empowerment of patients and citizens.

## Author contributions

The author confirms being the sole contributor of this work and has approved it for publication.

### Conflict of interest statement

SG-S is a consultant to the Royal Pharmaceutical Society and the Professional Records Standards Body. He has received no financial support from these or any other organizations to research and write this paper.
